# The Role of Interleukin-18, Oxidative Stress and Metabolic Syndrome in Alzheimer’s Disease

**DOI:** 10.3390/jcm6050055

**Published:** 2017-05-21

**Authors:** Johanna O. Ojala, Elina M. Sutinen

**Affiliations:** Neurology, Institute of Clinical Medicine, University of Eastern Finland, P.O. Box 1627, FI-70211 Kuopio, Finland; elina.siira@uef.fi

**Keywords:** interleukin-18, inflammation, oxidative stress, Alzheimer’s disease, metabolic syndrome, blood-brain barrier, BVR, DJ-1, DDAH, peroxiredoxin, enolase, 14-3-3, MMP14, Bcl-xL

## Abstract

The role of interleukins (ILs) and oxidative stress (OS) in precipitating neurodegenerative diseases including sporadic Alzheimer’s disease (AD), requires further clarification. In addition to neuropathological hallmarks—extracellular neuritic amyloid-β (Aβ) plaques, neurofibrillary tangles (NFT) containing hyperphosphorylated tau and neuronal loss—chronic inflammation, as well as oxidative and excitotoxic damage, are present in the AD brain. The pathological sequelae and the interaction of these events during the course of AD need further investigation. The brain is particularly sensitive to OS, due to the richness of its peroxidation-sensitive fatty acids, coupled with its high oxygen demand. At the same time, the brain lack robust antioxidant systems. Among the multiple mechanisms and triggers by which OS can accumulate, inflammatory cytokines can sustain oxidative and nitrosative stress, leading eventually to cellular damage. Understanding the consequences of inflammation and OS may clarify the initial events underlying AD, including in interaction with genetic factors. Inflammatory cytokines are potential inducers of aberrant gene expression through transcription factors. Susceptibility disorders for AD, including obesity, type-2 diabetes, cardiovascular diseases and metabolic syndrome have been linked to increases in the proinflammatory cytokine, IL-18, which also regulates multiple AD related proteins. The association of IL-18 with AD and AD-linked medical conditions are reviewed in the article. Such data indicates that an active lifestyle, coupled to a healthy diet can ameliorate inflammation and reduce the risk of sporadic AD.

## 1. Introduction

Alzheimer’s disease (AD), and particularly sporadic late-onset AD (LOAD), is already a major health issue globally, with AD incidence continually increasing. Partially it is explainable by aging and lengthened lifespan. A number of genetic factors and female gender are important contributors to AD, but even those cannot fully explain the worldwide rise of AD cases. The increasing incidence of LOAD indicates a significant role for environmental and lifestyle factors. Some medical conditions, partially related to lifestyle, increase AD risk, including obesity and type-2 diabetes (T2DM), where both chronic low-grade inflammation and oxidative stress (OS) are present. Chronic inflammation and OS increases lipid peroxidation and free-radical mediated cell death, with relevance to the etiology and course of many neurodegenerative diseases, including AD [1,2,3,4,5,6]. However, despite intensive investigation, the sequencing and interactions of the pathological events involved in development and progression of AD, including inflammation and OS, excitotoxicity and the accumulation of Aβ and neurofibrillary tangles (NFT), are still poorly understood. Nonetheless, it is widely believed that oxidatively modified proteins are intimately linked with the induction of NFT and Aβ pathology [7], with OS markers, such as 8-hydroxyguanosine and hydroxynonenal adducts, evident in the brain of early-stage AD [8].

Reactive oxygen species (ROS) are normally produced as by-products of oxidative metabolism, including during the course of normal mitochondrial respiration. However, they are also generated during pathological events, such as inflammation. Brain metabolism requires relatively large amounts of oxygen, with the oxidative burden further increasing during the course of normal aging [3,9]. Even during such normal aging, OS causes deterioration and mitochondria loss, from a shift in the oxidative-antioxidative balance, although these metabolic centers are protected by multiple defense systems. Such mitochondrial damage and loss can lead to cellular hypometabolism, with all these events increased in AD. Still, it is important to note that the increase in the oxidative-antioxidative balance is of some importance, as ROS have a role in many key cellular processes, including synaptic plasticity [10]. 

ROS can act as free radicals or they can react with other molecules to form free radicals. ROS include superoxide (O^2−^), hydroxyl radicals (OH˙), hydrogen peroxide (H_2_O_2_), and nitric oxide (NO) [2]. Neurons are especially sensitive to oxidants as their normal antioxidant content is low; and, further, neuronal membranes contain a large amount of oxidant sensitive polyunsaturated fatty acids [11]. Neurons also have high levels of iron, which can initiate harmful Fenton reactions, resulting in H_2_O_2_ reacting with Fe^2+^ to give rise to the highly reactive OH˙. Among free radical species, H_2_O_2_, released for instance in oxidative burst by microglia, seems to be a relatively stable compound that can diffuse freely across membranes. O^2−^ in turn is highly produced by mitochondria and is rapidly converted to H_2_O_2_, which can readily diffuse also within cells. These properties make H_2_O_2_ particularly toxic to the cells, which is further potentiated if converted to the highly reactive OH˙ [12]. H_2_O_2_ can also be detected in the rat brain at micromolar concentrations following ischemia/reperfusion [13]. Nevertheless, uncontrolled OS can trigger proapoptotic processes [2], but also induces endogenous antioxidant defenses, antiapoptotic signaling programs, and regenerative mechanisms [14], which contribute to the protein alterations typically evident in AD.

It is also important to note that the inflammatory and defense profile in humans differs from that of rodents [15,16]. Rodents do not normally develop AD, which is partially explained by their short lifespan, but it may also be due to their very efficient defense and clearance systems against microbes and toxins, generated by their natural diet and living habitat during the evolution. Therefore, the results of inflammatory studies in rodents may not be fully applicable to humans, though similarities exist. Nonetheless, chronic inflammation in the brain, like that detected in AD, is generally silent without fever and therefore differs from that of general systemic inflammation, reflected by different proportions of inflammatory factors and related molecules. Still, systemic inflammation can worsen symptoms in neurodegenerative diseases, including AD [17], and can also increase brain OS [18] as well as blood-brain barrier (BBB) permeability [19]. Aging increases inflammatory mediators and OS in the body [20], which increases cellular stress and causes epigenetic alterations, thereby linking aging to an increase risk of neurodegenerative diseases, especially if the antioxidant defense mechanisms are suboptimal and/or if risk alleles are present. 

Activated microglia, the principal defense cells in the central nervous system (CNS), and reactive astrocytes are the primary cellular sources of brain inflammation. Activated microglia and reactive astrocytes surround extracellular Aβ-plaques as well as dead or damaged neurons, including in AD [21,22,23]. Activated microglia release an array of inflammatory factors, including inflammatory cytokines and oxidative substances, as do reactive astrocytes, although generally to a lesser degree, versus microglia [24]. Yet, astrocytes are the most abundant cells in the brain, and when activated, can play an important, but poorly understood role in neurodegenerative diseases. Nevertheless, these various glia-derived inflammatory factors can generate ROS and ROS-driven OS in neurons, whereas an oxidative burst, a release of high ROS levels by activated microglia, increases exogenous OS. Therefore, neurons commonly suffer from collateral damages caused by these products. Further, aged neurons with poorly functioning defense and clearance systems can be more vulnerable.

ROS damages and increases the rigidity of all cell membranes, leading to modifications in proteins and lipids, as well as cell membrane receptors, which alters their function, intracellular signaling and clustering with other membrane receptors. For instance, many pro-inflammatory processes, including IL-18, activate the transcription factor, nuclear factor κ-light-chain-enhancer of activated B cells (NF-κB), leading to the induction of β-site amyloid precursor protein cleaving enzyme 1 (BACE-1) [25,26]. BACE-1 is an important initiator of amyloidogenic processing of amyloid precursor protein (APP). Therefore, heightened levels of OS and IL-18 can promote Aβ generation in neurons, with Aβ, in the presence of Cu^2+^ or Fe^3+^, producing H_2_O_2_ and OH˙ [27]. Aβ can also enhance OS generation by entering mitochondria, with the toxic actions of Aβ being at least partly prevented by antioxidants [28,29,30]. In addition, extracellular Aβ can promote microglia activation by increasing microglia respiratory burst activity and associated ROS. OS can induce expression of kinases, which hyperphosphorylate the microtubule associated protein tau [14], resulting in formation of NFTs. 

## 2. Proinflammatory Cytokine IL-18 and Hallmarks of Alzheimer’s Disease 

ROS can activate the nucleotide-binding domain, leucine-rich repeat -family and pyrin domain-containing protein 3 (NLRP3) inflammasome protein complex, composed of an intracellular sensor, such as a Nod-like receptor (nucleotide-binding oligomerization domain-like receptor), the precursor pro-caspase-1 and the adaptor protein apoptosis-associated speck-like protein containing a CARD (ASC). Inflammasome is primarily driven by caspase-1 [31], whereas activated caspase-1 mediates the cleavage, activation and secretion of inflammation, by enhancing IL-1β and IL-18 (formerly IL-1γ; interferon-γ (IFNγ) inducing factor (IGIF)), as well as cleaving several proteins and initiating an apoptotic pathway. IL-1β and IL-18 are mainly produced by microglia in the brain, but IL-18 is present also in reactive astrocytes and neurons [23]. IL-18 levels are elevated in AD brains [23], as well as in diseases that increase AD risk, including T2DM [32], obesity [33], and ischemic heart disease [34]. Further, physical and emotional stress can also elevate IL-18 levels [35,36], making IL-18 a susceptibility factor for depression [37], another condition associated with an increased risk of an array of neurodegenerative diseases and processes [38].

**Figure 1:**
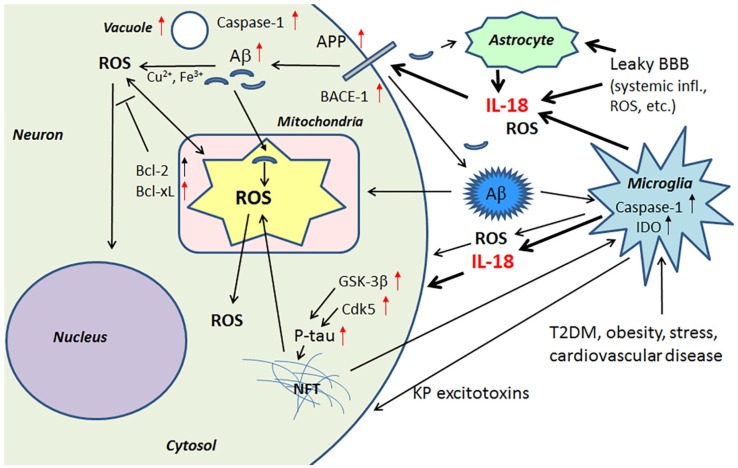
IL-18 has a role in Aβ generation and hyperphosphorylation of tau. Microglia and astrocytes, activated by multiple factors, produce IL-18 in the brain. IL-18 may also enter the brain through a leaky BBB, driven by systemic inflammation and ROS. IL-18 can enhance protein levels of APP, BACE-1 and Aβ, as well as GSK-3β and Cdk5, which are involved in tau hyperphosphorylation. IL-18 also increases vacuolization of neurons. Further, IL-18 seems to also increase ROS production in the cells. ROS in turn can activate caspase-1 and inflammasome system that can lead to further production of IL-18 and neuronal apoptosis. Red arrow, influenced by IL-18 [40,42].

IL-18 is a member of a rather large IL-1 gene family. In comparison to IL-1β, IL-18 expression seems to be higher in the AD brain, partly due to its longer half-life [23]. Although the normal function of IL-18 in the brain requires further investigation, it seems to play a role in CNS development and function as an up-stream regulator of brain immune and inflammatory processes [14,39]. Due to its proinflammatory nature, IL-18 is a potential enhancer of OS [40], which is also supported by the finding that IL-18 binding protein can ameliorate OS [41]. IL-18 can enhance Aβ production [42] and the kinases Cdk5 and glycogen synthase kinase-3β (GSK-3β), which are involved in the hyperphosphorylation of tau [14], caspase-1 regulation as well as many other neurodegenerative diseases linked proteins [40], and cellular vacuolization [42]. IL-18 can induce IFNγ, a significant driver of immune-inflammatory processes, as well as the primary inducer of indoleamine 2,3-dioxygenase (IDO). Activation of IDO redirects tryptophan metabolism towards the kynurenine pathway (KP), whereas activation of the KP is evident in depression, stress and many other medical conditions. The products of the KP can be excitotoxic, such as quinolinic acid, as well as diabetogenic, with such KP products proposed to contribute to AD neuropathology [43,44]. Therefore, enhanced or prolonged IL-18 levels, detected particularly in diseases related to lifestyle, can have an important role in AD pathogenesis. However, whether the protein changes are directly driven by IL-18 or mediated indirectly via OS, requires further investigation. The contribution of IL-18 to amyloidogenesis and NFT formation are indicated in Figure 1.

IL-18, directly or via OS, also modulates a number of key central processes, including antioxidative enzymes, such as peroxiredoxins 2, 3 and 6 (PRX2, PRX3, PRX6), protein deglycase DJ-1 (also known as Parkinson disease protein 7, Park7) and regulatory biliverdin reductase A (BVRA). Protein levels of dimethylarginine dimethylaminohydrolase 2 (DDAH2) can also be regulated by IL-18 [40]. In addition, IL-18 can moderately increase the hyperphosphorylation tau as well as modulate protein levels of the anti-apoptotic B-cell lymphoma-extra large (Bcl-xL) [42] and B-cell lymphoma 2 (Bcl-2) family of proteins. Bcl-xL is a protective transmembrane molecule that can be present in mitochondria, where it is protective against Aβ neurotoxicity [45]. Interestingly, Bcl-xL and Bcl-2 can also enhance dopaminergic neuron generation from neuronal stem cells as well as modulate the differentiation of immortalized human neural stem cells [46]. However, it is important to note defense system activation may vary depending on neuronal age.

### 2.1. Peroxiredoxins and Protein Deglycase DJ-1 and Their Role as Antioxidants in the Brain

The multifunctional antioxidant thioredoxin-dependent PRXs form a family (PRX1-6), with members that are differentially localized intracellularly, with additional variation across different cell types [47]. In general, PRXs protect cells against OS, such as ROS-mediated DNA-fragmentation. PRXs also regulate intracellular signaling cascades that utilize H_2_O_2_ as a second messenger molecule, as well as cell proliferation. PRX2 is expressed solely in neurons [1], and its deficiency makes neurons highly sensitive to H_2_O_2_, which commonly leads to cell death. PRX2 can also interact with presenilin-1 (PS-1), as can the anti-apoptotic Bcl-2 and Bcl-xL proteins. These interactions may regulate processing of APP to Aβ and protect against neuronal apoptosis [48,49]. IL-18 reduces protein levels of PRX2 [40], which may contribute to the neuronal damage evident in AD and T2DM.

Levels of mitochondrial PRX3 are decreased in the AD brain and leakage may explain this reduction since mitochondria are commonly damaged in AD, due to multiple causes [1]. However, in healthy young neuron-like cells, IL-18 was able to increase the expression of PRX3 [40]. In aged neurons, the impact of IL-18 can be different; with IL-18 facilitating the modifications of PRX3 protein, leading to its inactivation [50] or increasing its release. As well as reducing H_2_O_2_, PRX6 can also reduce short chain organic fatty acids and phospholipid hydroperoxides, indicating the importance of PRX6 in phospholipid turnover [51]. In AD, PRX6 is increased in astrocytes as well as in diffuse and neuritic plaques [52]. Like Aβ production, PRX6 is inducible by IL-18 [40,42], and may play a role in the regulation of Aβ aggregation. Enhanced PRX6 expression is also linked with increased levels of matrix metalloproteinase 9 (MMP9) and the urokinase-type plasminogen activator (uPA) [53]. Both uPA and tissue-type plasminogen activator (tPA) convert plasminogen to the active protease plasmin, which can degrade Aβ [54] and regulate MMP levels [55]. 

**Figure 2:**
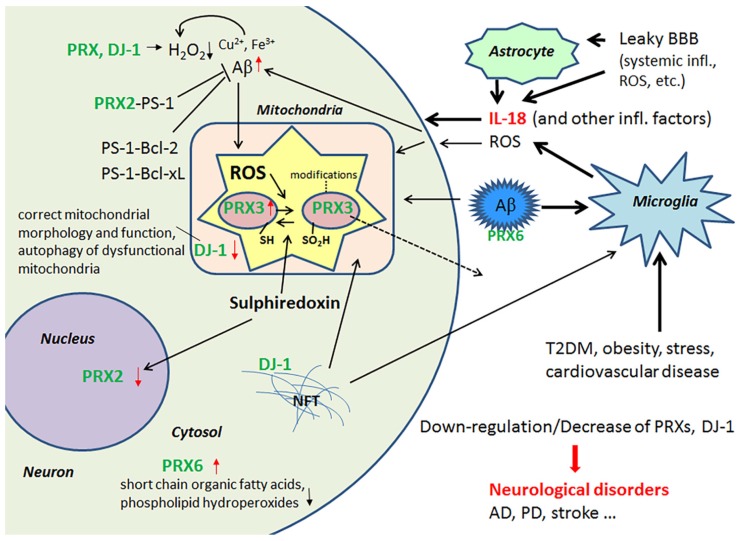
PRXs and DJ-1 in regulation of ROS and Aβ. Some of the PRXs are induced by IL-18, either directly or indirectly, whereas DJ-1 is down-regulated by IL-18 in neuron-like cells. Lack or inactivation of these ROS modulators and mitochondria protectors can lead to neurodegeneration. Red arrow, influenced by IL-18 [40,42].

IL-18 down-regulates protein levels of an atypical PRX-like peroxidase DJ-1 [34], which scavenges H_2_O_2_ and functions as a redox-sensitive chaperone [56,57]. DJ-1 also regulates oxidant status via the modulation of Cu/Zn-superoxide dismutase-1 levels [56,58]. However, DJ-1 is also crucial to the regulation of mitochondrial morphology and function, as well as in the autophagy of dysfunctional mitochondria, indicating why the loss of functional DJ-1 commonly leads to neurodegeneration [59]. For instance, it is reduced in the CNS of patients with Parkinson’s disease (PD), but on the other hand, it seems to be present in tau inclusions [60]. Interestingly, DJ-1 is also reduced in pancreatic islets of T2DM patients. However, under non-diabetic conditions, DJ-1 expression increases in islets over aging, preventing an increase in ROS levels. DJ-1 preserves mitochondrial integrity and physiology, as shown in both mice and humans [61]. DJ-1-deficient mice develop glucose intolerance and reduced β cell area, as they age or gain weight, indicating that DJ-1 plays a key role in glucose homeostasis and prevents the development of T2DM [61]. IL-18 regulation of PRXs and DJ-1 are summarized in Figure 2.

### 2.2. Biliverdin/Bilirubin-System, Dimethylarginine Dimethylaminohydrolase 2 and Nitrosative Stress

Evolutionarily conserved, multifunctional BVR and heme oxygenase (HO) are crucial in the defense against OS. BVR is expressed in all tissues; BVRA is dominant in adults and BVRB in the fetus [62]. HO is required to cleave the heme ring to form biliverdin (BV), whereas soluble BVR converts BV to lipophilic bilirubin (BR), which is a potent antioxidant [63]. However, both BR and BV function as strong scavengers of peroxyl radicals, with BR also effective against lipophilic ROS [64]. BR also neutralizes NO radicals by forming NO-BR [65], which inhibits ROS reaction with NO and generation of extremely harmful compounds. BVRA has a regulatory role in AD [66], whereas IL-18 increases can BVRA levels in AD [40]. However, several factors, including BV and phosphorylation, can also regulate BVRs [66,67]. BVR, a member of the insulin receptor substrate family, also has other, less well characterized roles in cells, functioning as a Ser/Thr/Tyr-kinase and as a transcription factor, as well as also being involved in cell-signaling, which is mediated by protein kinase C (PKC), mitogen activated protein kinase (MAPK) and phosphatidylinositol 3-kinase (PI3K) [62,67,68,69]. Therefore, IL-18 or other factors increasing BVR are also likely to modulate other neuronal antioxidants and intracellular signaling pathways, as well as transcription per se.

**Figure 3:**
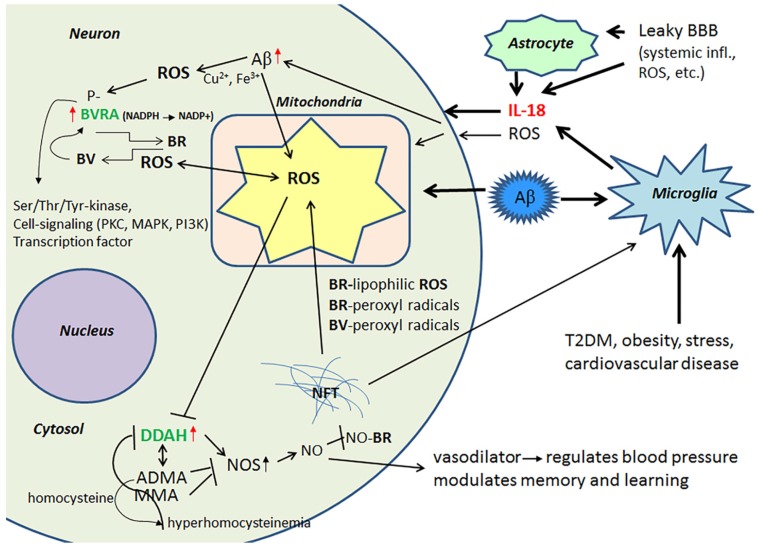
BVR/Biliverdin/bilirubin system and DDAH. IL-18 is a regulator of BVRA, which maintains the functional antioxidative BV/BR-system. BVRA is activated by phosphorylation [66]. BVRA also influences cell signaling and transcription. Dysregulation of DDAH can lead to hyperhomocysteinemia, a risk factor for instance for AD and atherosclerosis. Red arrow, influenced by IL-18 [40,42].

Protein levels of DDAH are increased in the frontal lobes of AD patients, which can partially be driven by IL-18 [40]. DDAH is involved in the regulation of cellular methylarginine concentration by hydrolyzing N(G), N(G)-dimethyl-l-arginine (ADMA) and N(G)-monomethyl-l-arginine (MMA). Both ADMA and MMA act as inhibitors for nitric oxide synthase (NOS) [70,71]. When these endogenous NOS inhibitors are catabolized by DDAH, NOS activity and NO production can be increased resulting in nitrosative stress, leading to neuronal and synaptic dysfunction. However, NO also has a vital role in synaptic plasticity, and is therefore important to the modulation of memory and learning [70]. In addition, the ADMA/DDAH pathway regulates angiogenesis [72], whilst NO functions as a vasodilator, thereby regulating blood pressure [73]. However, the activity of DDAH gets impaired by OS, inflammatory cytokines and/or hyperhomocysteinemia leading to accumulation of ADMA. The byproduct of ADMA synthesis is homocysteine, with hyperhomocysteinemia being a risk factor for cardiovascular disease, stroke and dementia [74,75]. Figure 3 summarizes the impact of IL-18 on BVRA and DDAH.

### 2.3. IL-18, Oxidative and Nitrosative Stress and Matrix Metalloproteinases as Regulators of the Blood-Brain Barrier

Activated MMPs regulate BBB integrity, having a role in the digestion of tight junctions and BBB basement membrane proteins and are therefore critical contributors to different brain diseases [76,77]. Although MMPs play a crucial role in remodeling processes in developing and regenerating tissues, including neuronal networks in the brain [78,79], they also cleave cell surface molecules and soluble factors, such as chemokines, cytokines and their receptors [79]. Consequently, MMPs have an important role in fine-tuning an array of cellular processes, including those involved in inflammation [79,80,81,82]. MMPs also activate neuroinflammatory pathways [82,83].

**Figure 4:**
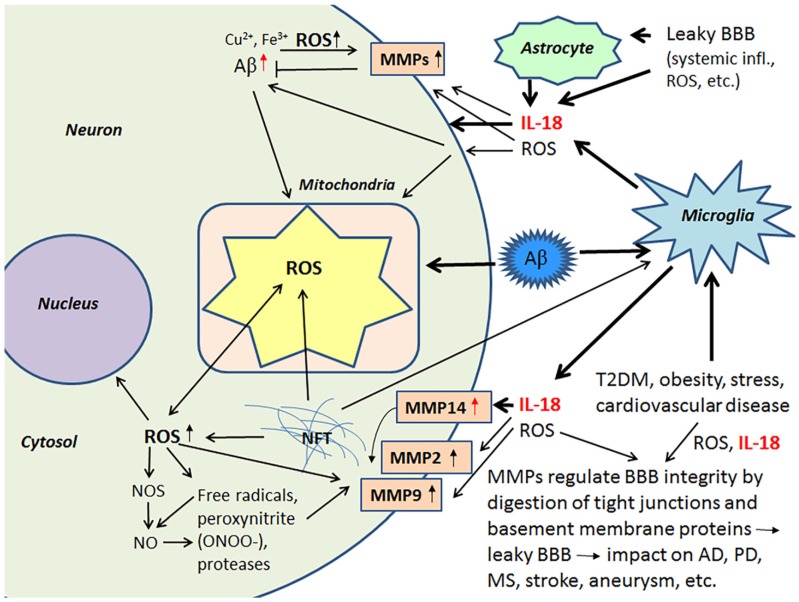
The multiple roles of MMPs in neurodegeneration. ROS are powerful inducers of MMPs, and induction can lead to leakage in BBB with harmful consequences. On the other hand, MMPs can degrade Aβ and reduce ROS production. Red arrow, target regulated by IL-18 [40,42].

ROS are key mediators of increased BBB permeability [84]. Free radicals, peroxynitrite (ONOO-) and proteases activate MMPs, including MMP9 and MMP2, which are important in the digestion of the endothelial basal lamina leading to BBB permeability [85,86]. IL-18 also induces MMP2 and MMP9 [87,88], as well as MMP14 (also called a membrane type-1 MMP (MT1-MMP) [40]. Several MMPs, including pro-MMP2 are activated by MMP14 [89,90]. Among other functions MMP2, MMP9 and the soluble MMP14 form, to some degree, contribute to the degradation of Aβ [86,91,92]. In AD patients, MMP14 levels are increased in the frontal lobe versus the occipital lobe, although to a lesser degree versus healthy controls [40]. The influence of IL-18 on some of the MMPs is shown in Figure 4. 

Since MMPs contribute to ROS and inflammation-induced BBB dysfunction and leakage, MMPs can also promote the progression of several CNS disorders, including AD, stroke, PD, multiple sclerosis and cerebral aneurysms [83,93]. Ischemic stroke is a major risk factor for not only vascular dementia, but also AD, whilst obesity is an independent risk factor for an array of medical conditions, including stroke. Stroke induces OS/mitochondrial dysfunction, inflammatory responses, micro RNA alterations and marked changes in brain proteins [94], that are likely to contribute to the array of pathophysiological changes evident in AD. Such data highlights the importance of the maintenance of the BBB. BBB permeability and brain microvascular MMP9 expression are markedly increased in obese mice, indicating that obesity potentiates brain microvascular disruption, including in the pathophysiological underpinnings of stroke susceptibility [95]. Further, a high-fat diet enhances the brain infarct volume, as well as increasing brain edema, BBB damage, higher hemorrhagic transformation rate, greater hemorrhagic volume and worse neurological function, as evident in murine models. High-fat diet also raises MMP9 activity in ischemic and non-ischemic brain tissues [96].

## 3. Metabolic Syndrome

Late-onset AD (LOAD) represents 90–95% of all AD cases, with increasing data indicating that both LOAD and AD are related to metabolic diseases and thereby closely linked to vascular and related factors. Such factors include total cholesterol and other lipid parameters, as well as hypertension and metabolic alterations, such as glucose utilization impairments in the brain, insulin responsiveness and energy metabolism. Metabolic syndrome (MetS) covers all these conditions, namely abdominal obesity, increased blood pressure, elevated fasting plasma glucose, and low high-density lipoprotein (HDL) levels or high serum triglyceride levels. When these parameters occur together, there is an increased risk of cardiovascular disease, stroke and T2DM, as well as cognitive impairment and dementia, with the most important risk factor being hyperglycemia [97,98].

Factors involved in MetS generate chronic systemic low-grade inflammation, “metaflamation”, which interferes with adipose tissue homeostasis and insulin signaling. In the brain, insulin and insulin-like growth factor (IGF) signaling are involved in synaptic maintenance, neuroprotection and neuronal growth [99,100]. During normal insulin signaling, insulin degrading enzyme (IDE) is essential, with IDE also contributing to Aβ clearance. IDE is inhibited by free fatty acids (FFAs), which are elevated in obesity. Inhibition of IDE increases Aβ levels and facilitates its deposition, with Aβ deposits triggering OS, which can cause an array of damaging events, including to the sensitive layer of the endothelial brain vasculature. Hypertension also causes functional and morphological changes in the cerebral vascular system, including vessel remodeling, endothelial dysfunction, BBB damages and cerebral microbleeds, which increases neuronal death and can eventually lead to the onset or progression of cognitive impairment [98]. Many of these changes are mediated by MMPs.

Partly as a consequence of mitochondrial loss or damage due to Aβ and OS, AD also shows evidence of glucose hypometabolism. MetS and diabetes affect mitochondrial dynamics, suggesting mitochondria as critical sites in the progression of AD. Mice fed a high fat diet develop obesity, lower glucose and insulin tolerance, and elicit insulin resistance in the brain, versus mice fed with a normal diet. The brains of these mice also exhibit biochemical changes associated with increased Aβ deposition and NFT formation, as well as decreased synaptic plasticity [101]. Sucrose-treated mice also show mitochondrial abnormalities, oxidative imbalance and a significant increase in Aβ levels as well as increases in phosphorylated tau (pTau) levels [102]. Overall, as well as mutations in mitochondrial DNA and the DNA repair response, and exposure of environmental toxins, it is clear that a high-calorie intake and glucolipotoxicity can lead to mitochondrial dysfunction and increased ROS production with multiple negative influences in the brain [103,104]. 

### 3.1. IL-18, Enolases and Multiple Functioning 14-3-3

Among the multiple activators, the saturated FFA, palmitate, induces the activation of the NLRP3 inflammasome protein complex. In this activation process, FFA palmitate signals through an adenosine monophosphate activated protein kinase (AMPK)-autophagy-mitochondrial ROS pathway [105], with the activation of NLRP3 leading to the maturation of caspase-1 and the processing of its substrates, including IL-18. In general, the inflammasome is a complex that modulates infection and inflammation, with consequences for atherosclerosis, T2DM, gout, obesity and an array of other medical conditions. Another important feature of inflammation, and the inflammasome specifically, is that the activation process has a profound effect on aerobic glycolysis (the “Warburg effect”), commonly involved in cancer [106,107]. ROS producing oxidative phosphorylation (the ‘inverse Warburg Effect’) in turn is linked to AD and PD. Nonetheless, IL-18 influences glucose metabolism by its ability to increase glycolytic γ-enolase (ENOG) levels and decrease α-enolase (ENOA) levels [40]. In general, ENOs form homo- or heterodimers, and they are associated with hypoxia, ischemia and AD [108,109].

ENOA decline can increase ROS, mainly generated through the nicotinamide adenine dinucleotide phosphate (NADPH) oxidase pathways, as well as autophagy and catabolic pathway adaptations, which together can affect many cellular processes, including cell growth and senescence [107]. In mild cognitive impairment and AD, upregulation of ENOA seems to be a prominent feature [108]. However, in AD, protein modifications, such as oxidization, glycosylation, glutathionylation or nitration, are common, with ENOA being a common target. These modifications can lead to its catalytic inactivation, and may partly explain the ENOA increase in AD [110,111,112,113]. The role of IL-18 in this process is not known. ENOA is indirectly involved in Aβ metabolism, due to its additional role as a plasminogen receptor on the surface of several cell types [114]. When plasminogen interacts with ENOA, processing of plasminogen to plasmin is increased, mediated by tPA or uPA [115]. ENOA’s catalytic inactivation by modifications likely alters also its role as a plasminogen receptor [116], with consequences for Aβ degradation and neuronal survival, but also for extracellular matrix remodeling.

ENOG levels are increased significantly in an array of medical conditions, including Creutzfeldt-Jakob disease, cerebral trauma, brain tumors and cardiovascular disease. Glycolytic ENOG also has additional roles, including functioning as a neurotrophic-like factor, leading to neuronal growth, regeneration, differentiation and survival. This occurs after its translocation and binding to the plasma membrane [117,118,119]. The neurotrophic activity of ENOG is modulated by its C-terminal peptide, which activates the PI3K/Akt and MAPK/extracellular signal-regulated kinase (ERK) signaling pathways [119]. Cathepsin X is involved in cleaving the C-terminal peptide of ENOG and ENOA, with such cleavage impairing the survival and neuritogenesis of neuronal cells. Interestingly, the activation of Cathepsin X increases in an age-dependent manner and is also associated with Aβ-plaques [120,121]. 

**Figure 5:**
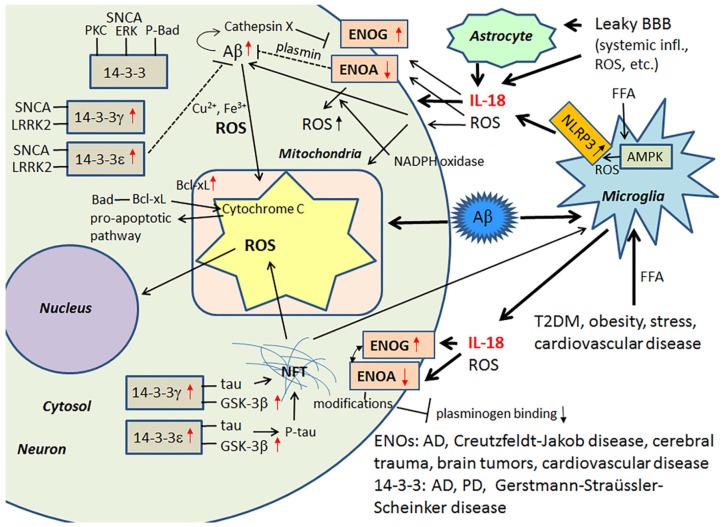
ENOs and 14-3-3 in neurodegeneration. Glycolytic ENOs also have a role in degradation of Aβ, mediated by plasmin. 14-3-3 proteins can regulate aggregation of SNCA [128,129], as well as influence hyperphosphorylation of tau [125]. ROS, generated for instance by Aβ and NFT, can deteriorate mitochondria leading to release of cytochrome C and initiation of pro-apoptotic pathway. As the 14-3-3 proteins, SNCA can interact with PKC, ERK and P-Bad [123]. Free Bad can interact with Bcl-xL, which leads to the release of cytochrome C from mitochondria and the initiation of the pro-apoptotic pathway [132]. Red arrow, influenced by IL-18 [40,42].

14-3-3 proteins belong to a highly conserved protein family of ubiquitous cytoplasmic chaperones that play important role in metabolism, signal transduction, intracellular trafficking, cell cycle control and apoptosis [122,123]. IL-18 can increase expression at least some of these multifunctional 14-3-3 isoforms, including 14-3-3γ and -ε, which are commonly affected in neurodegenerative diseases. These 14-3-3 isoforms can also bind to GSK-3β [14,40], which is increased in AD and MetS [98,124], with the simultaneous binding to tau and GSK-3β promoting tau phosphorylation, which can eventually lead to the formation of NFTs [125]. 14-3-3ε is also indirectly involved in Aβ metabolism [126], and it is a part of the prion protein amyloid deposits of Gerstmann-Straüssler-Scheinker disease [127]. Further, both 14-3-3ε and 14-3-3γ have interactions with leucine-rich repeat Ser/Thr-protein kinase 2 (LRRK2), which is also detectable in α-synuclein (SNCA) positive Lewy bodies and in Hirano bodies in AD [128,129]. Interestingly, PD related SNCA shares physical and functional homology with 14-3-3 proteins, and both 14-3-3γ and -ε can interact with SNCA and prevent inclusion formation [130]. SNCA also binds to some other proteins known to associate with 14-3-3, including PKC, ERK and the pro-apoptotic Bcl-2-associated death promoter (Bad) [123]. In general, 14-3-3 proteins can interact with phosphorylated Bad and sequester Bad to the cytosol [131]. Free Bad can interact with Bcl-xL, leading to the release of cytochrome c from mitochondria and the initiation of the pro-apoptotic pathway [132]. Neuronal apoptosis and damage, generated by ischemic cerebral infarction, can be reduced by 14-3-3ε [133]. Figure 5 summarizes the impact of IL-18 on the above described targets.

## 4. The Effect of Lifestyle and Diet on the Genesis/Progression of Alzheimer’s Disease

Dementia is also associated with low education and low levels of book reading, with other lifestyle activities, such as learning new things and having an active social network, able to delay dementia onset, most likely by maintaining or improving the neural plasticity and synaptic network. However, these lifestyle factors do not seem to significantly influence the expression of AD pathophysiology [134,135]. Another mechanism for protection might also be the reduction of unpleasant, long-lasting stress by different kind of hobbies. Harmful stress reaction also involves IL-18 and KP pathway activation [38,44].

Physical inactivity is a risk factor for AD, as well as the development of MetS and cardiovascular diseases. Regular physical exercise enhances metabolism and circulation, including in the brain, with resultant improvements in neurogenesis. Increased cardiorespiratory fitness is also associated with improved memory performance and reduced hippocampal atrophy. In AD patients, exercise improves cognitive function, decrease neuropsychiatric symptoms and slow the decline in activities of daily living (ADL). Therefore, regular physical exercise can be recommended as a prevention strategy for AD as well as one type of treatment for pre-clinical and late stage AD. Aerobic exercise in early AD is particularly associated with benefits in cognitive functioning [136,137,138].

Among people with MetS, IL-18, in comparison to other pro-inflammatory cytokines, seems to be the best marker for inflammation [139]. High-intensity aerobic interval training in turn significantly reduces serum IL-18 levels in patients with MetS, but, for some reason, not TNF-α, IL-6, insulin or high sensitive C-reactive protein (hsCRP) levels [139]. As in muscles, neurons seem to respond to aerobic exercise, but also food deprivation, by activating signaling pathways, including Ca^2+^, cAMP response element-binding protein (CREB), peroxisome proliferator-activated receptor-γ coactivator 1-α (PGC-1α) and NF-κB. These signaling pathways can stimulate mitochondrial biogenesis and cellular stress resistance. Neurons counteract the damaging challenges also by up-regulating antioxidant defenses, autophagy/mitophagy and DNA repair [140]. However, too exhausting endurance training can decrease or deplete antioxidant levels, which is likely due to the increased oxygen metabolism and increased ROS formation [141]. Aging may enhance that effect, and therefore rest is required in recovery process, particularly in old age, to gain the benefits of exercise.

In addition to physical activity, good nutrition throughout life can also prevent or delay chronic diseases and disabilities in old age, including AD [142,143,144]. Endogenous and exogenous antioxidants, including those originating from nutrition, protect cellular structures against OS. Therefore, a diet rich in vitamins, polyphenolic compounds and polyunsaturated fatty acids and low in saturated fatty acids has been recommended. These nutrients are modifiable, bioactive and exert potent antioxidant and anti-inflammatory activities. They also have the ability to influence biochemical and biological processes that can preserve a healthy brain status and support brain plasticity. For example, neuronal plasticity improvement by omega-3 intake has been found to be mediated by the upregulation of brain-derived neurotrophic factor (BDNF) [145,146]. However, cooking procedures can modify the original dietary content, including the loss of healthy nutrients and the formation of different kind of toxins and advanced glycation end products (AGEs). The compounds are adsorbed at intestinal levels and can contribute to the aging process and neurodegeneration [144,147].

### 4.1. Lipid Rafts and Their Composition

Important targets for OS and nitrosative stress mediated damage and function alterations are cell membranes with their variety of protein receptors, commonly harbored in lipid rafts. Nutrition influences the composition and function of cell membranes, including lipid rafts. Lipid rafts are microdomains, composed of cholesterol and glycosphingolipids, which are crucial components and regulators of cellular signaling and neurotransmission [148]. Activities of BACE-1 and PS-1 are regulated by membrane lipids and raft formation [149,150]. OS in turn can up-regulate PS-1 in lipid rafts in neuronal cells, and this seems to be mediated through up-regulation of PS-1 transcription [150]. 

Elevated total cholesterol in midlife has been associated with increased risk of dementia in later life, whereas the cholesterol oxidation metabolite 27-hydroxycholesterol (27-OHC) has been shown to cause AD-like pathology in cell culture models [151]. Further, lipids, but not proteins, extracted from oxidized low density lipoprotein (oxLDL) have been shown to be more cytotoxic than oxLDL. Interestingly, 27-OHC and total lipids from LDL and oxLDL can independently increase Aβ production [149]. Whether the influence is mediated through modified lipid rafts harboring PS-1 or BACE-1, remains to be determined.

A significant decline in the omega-3 polyunsaturated fatty acid docosahexaenoic acid (DHA) seems to be associated to both natural aging and AD [152]. Although DHA and eicosapentaenoic acid (EPA) can integrate into lipid rafts, DHA has a greater tendency to incorporate than EPA. The incorporation leads to disruption of lipid raft domain organization [153], and likely also alterations in PS-1 and BACE-1 activities. Interestingly, very long omega-3 polyunsaturated fatty acids, as well as Mediterranean and Nordic diets, can also reduce serum levels of IL-18 in elderly atherosclerosis high-risk men [154,155].

## 5. Summary, Conclusions and Future Directions

**Figure 6:**
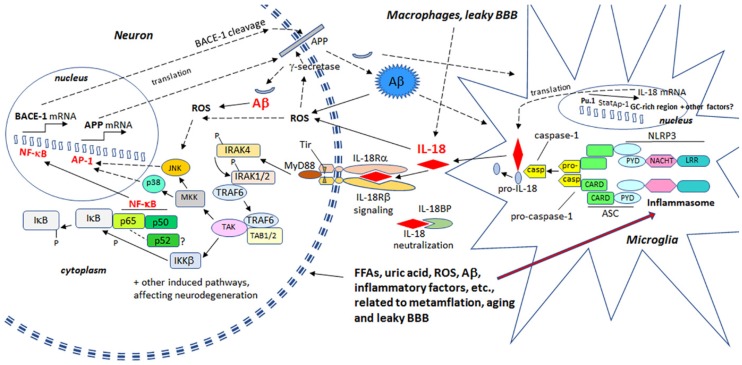
IL-18 enhances BACE-1 and APP production leading to increase in Aβ levels. Microglia and reactive astrocytes are the main producers of IL-18 in the brain. Expression of IL-18 is regulated by transcription factor PU.1, and the other regulatory areas include GC-rich region, AP-1 and STAT binding sites [160,161]. IL-18 can enhance transcription factors NF-κB and AP-1 [162]. Angiotensin II, implicated in atherosclerosis, is one inducer of IL-18 expression [162], whereas numerous inflammasome activators lead to activation of caspase-1 and increase in secretion of bioactive IL-18. NF-κB has a role in BACE-1 in regulation [25,26], whereas expression of APP is modulated by AP-1 [163]. Both NF-κB and AP-1 are also inducible by ROS. AP-1, activator protein 1 transcription factor; IKK, inhibitor of κB kinase; IRAK, Interleukin-1 receptor-associated kinase; IL-18BP, IL-18 binding protein; JNK, c-Jun N-terminal kinase; LRR, leucine-rich repeat; MKK, mitogen-activated protein kinase kinase; MyD88, myeloid differentiation primary response gene 88; NACHT, NAIP (neuronal apoptosis inhibitor protein), C2TA (class 2 transcription activator of the major histocompatibility complex, HET-E (heterokaryon incompatibility), TP1 (telomerase-associated protein 1); NLRP, NLR (nucleotide-binding domain, leucine-rich repeat) family, pyrin domain containing; Pu.1, PU-box (a purine-rich DNA sequence (5′-GAGGAA-3′)) binding transcription factor (SPI1; Spi-1 proto-oncogene); PYD, pyrin domain; Stat, signal transducer and activator of transcription; TAB, TGF-β (transforming growth factor-β)-activated kinase and MAP3K7-binding protein; TAK, mitogen-activated protein kinase kinase kinase (MAP3K); Tir, Toll/interleukin-1 receptor homology domain; TRAF6, TNF (tumor necrosis factor) receptor associated factor.

**Figure 7:**
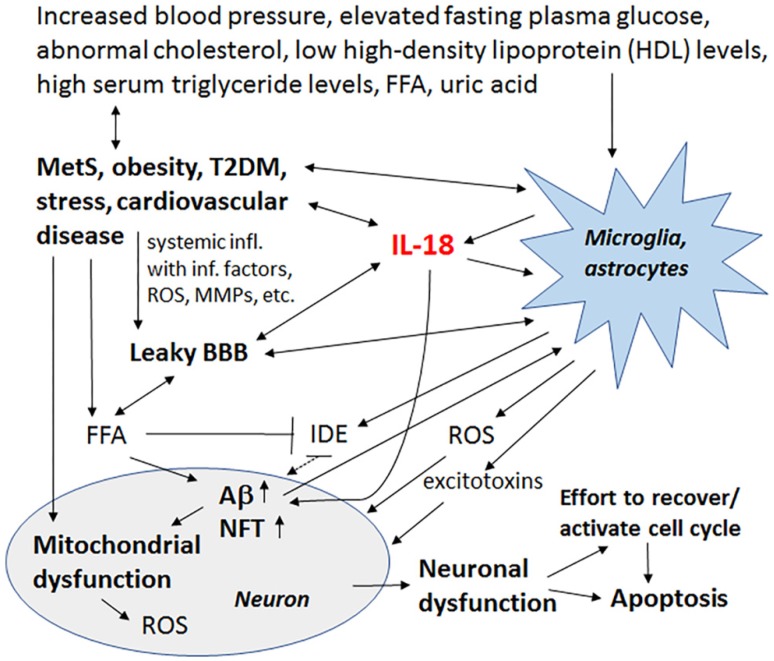
MetS and its role in neurodegeneration. IL-18 and OS, linked to AD and conditions that are associated to MetS, have multiple impacts on targets related to these conditions. Further, hallmarks of MetS have multiple harmful influences on microglia, astrocytes and neurons, which can lead to increase in Aβ production and NFT formation, and eventually, neuronal loss. Crucial players in this process can be IL-18 and ROS. However, inflammatory changes caused by MetS are often called metaflamation, which is a feverless inflammation. The term metaflamation also draws intention to multiple environmental and lifestyle issues, which is mainly generated by an excess of nutrients and metabolic tissue-driven cytokines. The chronic low grade inflammation often causes fatigue, cognitive impairment, sleep alterations, chronic pain and depression, frequently evident in AD as well as in MetS and related disorders.

Increased IL-18 levels (Figure 6), linked to MetS, obesity, cardiovascular diseases, T2DM, depression and stress (Figure 7), can have multiple impacts on neurons, with similarities in these conditions to those detected in AD. For instance, IL-18 increases Aβ production, as well as expression of Cdk5 and GSK-3β, which are involved in tau hyperphosphorylation. IL-18 also induces mitochondrial stress and OS in neurons, either directly or indirectly, including via Aβ (Figure 1). Induction of OS by IL-18 is supported by the increase in antioxidative enzymes PRX3 and PRX6 (Figure 2), BVRA (Figure 3), as well as the generally neuroprotective 14-3-3 proteins (Figure 5) in neurons, as a defense mechanism. In MetS, BVRA protects against hepatic steatosis by inhibiting GSK-3β. BVRA knock-out mice in turn exhibit increased plasma glucose and insulin levels and decreased glycogen storage [156]. Reduction of DJ-1 by IL-18 suggests mitochondrial stress (Figure 2), whereas enhanced DDAH expression indicates increased nitrosative stress (Figure 3). DJ-1 is also reduced in the pancreatic islets of T2DM patients [61], and DDAH, as well as ADMA, has also been closely linked with the development of T2DM and obesity [157]. IL-18 and ROS can also regulate BBB function through enhancing the expression of several MMPs, including MMP9 (Figure 4). Brain microvascular MMP9 expression and BBB dysfunction with associated increased BBB permeability, are also markedly increased in obese mice, thereby promoting the extravasation of inflammatory factors and ROS in the brain, with likely impacts on AD progression [95]. Decreased or inactivated ENOA and 14-3-3ε in neurons by IL-18 can also indirectly lead to increase in Aβ accumulation (Figure 5). These multiple changes may also lead to activation of caspase-1 and eventually neuronal death. The harmful effects of pro-longed high IL-18 and OS levels are likely to be more severe in aged and variably-stressed neurons, with different defense and clearance systems concurrently less effective. A number of AD susceptibility genes are likely to interact with these processes, at both central and systemic sites. Chronic inflammation can also generate epigenetic alterations. 

In people with MetS, high-intensity aerobic interval training can significantly reduce serum IL-18 levels [139]. Regular physical training also reinforces antioxidative processes during recovery, thereby reducing OS and optimizing anti-inflammatory defenses. Exercise also improves endothelial function, potentially improves brain capillarization, and can further counteract dyslipidemia. Importantly, regular physical activity can also induce neurogenesis [158]. Healthy diet containing omega-3 fatty acids and antioxidants can also be effective in reducing IL-18 levels [154,155]. Generally, bioactive IL-18 is regulated by an activated inflammasome-system, as well as IL-18 binding protein and IL-18 receptors (Figure 6), whereas physical activity, nutrition and stress can play an important role in its transcriptional and translational regulation, as well as in its activation or inactivation. Prolonged IL-18 elevation can damage neurons, both directly and indirectly, including in diabetic neuropathy. IL-18 may also be involved in weight regulation [159], as yet only shown in murine models. Further, the role of IL-18 in the regulation of OS and its influence on mitochondrial functioning requires further investigation, as does the transcriptional regulation and signaling pathways of IL-18. IL-18 may also play some role in PD.

In conclusion, a lifetime of moderate exercise, in combination with a healthy diet containing enough antioxidative components and omega-3, as well as an intellectually challenging lifestyle may be effective in the prevention of LOAD, with efficacy that is at least partially mediated via the central and systemic regulation of IL-18 and ROS. 

## Abbreviations

Aβ, Amyloid-β; AD, Alzheimer’s disease; ADL, activities of daily living; ADMA, N(G),N(G)-dimethyl-l-arginine; AGE, advanced glycation end product; APP, Amyloid-β precursor protein; AMPK, adenosine monophosphate (AMP)-activated protein kinase; ASC, apoptosis-associated speck-like protein containing a CARD (caspase recruitment domain containing; BACE-1, β-site APP cleaving enzyme 1; Bad, Bcl-2-associated death promoter; Bcl-2, B-cell lymphoma 2; Bcl-xL, B-cell lymphoma-extra large protein; BDNF, brain derived neurotrophic factor; BVR, biliverdin reductase; BR, bilirubin; BV, biliverdin; Cdk5; cyclin dependent kinase-5; CNS, central nervous system; CREB, cAMP response element-binding protein; DDAH, N(G), N(G)-dimethylarginine dimethylaminohydrolase; DHA, docosahexaenoic acid; DJ-1, Protein deglycase, Parkinson disease protein 7; ENOA, enolase α; ENOG, enolase γ; EPA, eicosapentaenoic acid; ERK, extracellular signal-regulated kinase; FFA, free fatty acid; GSK-3β, glycogen synthase kinase-3β; HO, heme oxygenase; 27-OHC, 27-hydroxycholesterol; IDE, insulin degrading enzyme; IFNγ, interferon-γ; IGIF, IFNγ inducing factor; KP, kynurenine pathway; MAPK, mitogen activated protein kinase; LOAD, late-onset AD; MetS, Metabolic syndrome; MMA, N(G)-monomethyl-l-arginine; (MT1)-MMP, (membrane-type-1) matrix metalloproteinase; NADPH, nicotinamide adenine dinucleotide phosphate; NF-κB, nuclear factor κ-light-chain-enhancer of activated B cells; NFT, neurofibrillary tangle; NLRP, NLR (nucleotide-binding domain, leucine-rich repeat) family, pyrin domain containing; Nod-like receptor, nucleotide-binding oligomerization domain-like receptor; NOS, nitric oxide synthase; OS, oxidative stress; oxLDL, oxidized low density lipoprotein; PD, Parkinson’s disease; PGC-1α, peroxisome proliferator-activated receptor-γ coactivator 1-α; PI3K, phosphatidylinositol 3-kinase; PKC, protein kinase C; PRX, peroxiredoxin; PS-1, presenilin 1; ROS, reactive oxygen species; α-synuclein, SNCA; T2DM, type-2 diabetes mellitus; tPA, tissue-type plasminogen activator; uPA, urokinase-type plasminogen activator.
